# Megalin/lipoprotein receptor-related protein 2 autoimmunity and kidney disease

**DOI:** 10.1093/ckj/sfz171

**Published:** 2020-03-19

**Authors:** Maria V Perez-Gomez, Maria D Sanchez-Niño, Alberto Ortiz

**Affiliations:** 1 IIS-Fundacion Jimenez Diaz, School of Medicine, Universidad Autonoma de Madrid, Madrid, Spain; 2 Fundacion Renal Iñigo Alvarez de Toledo-IRSIN and REDINREN, Madrid, Spain

**Keywords:** acute interstitial nephritis, CD69, Fx1A, LRP2, megalin, membranous nephropathy, proteinuria

## Abstract

In this issue of *Clinical Kidney Journal*, Gamayo *et al.* describe two cases of anti-low-density lipoprotein receptor-related protein 2 (LRP2) nephropathy. This is a recently described entity that has features of both tubulointerstitial disease and segmental membranous nephropathy. The originality of the present report consists of the association of a disease thought to be rare (only 13 in prior described patients, 11 in the past year) with B-cell lymphoproliferative disease. Together with the finding of a third case among 224 elderly patients studied, this raises the issue of the underdiagnoses of LRP2 nephropathy, on top of the potential association to B-cell malignancy. We now put these findings in context within the wider frame of autoimmunity against megalin/LRP2 and related antigens such as Fx1A and CD69.

In this issue of *Clinical Kidney Journal* (*ckj*), Gamayo *et al*. describe two cases of anti-low-density lipoprotein receptor-related protein 2 (LRP2) nephropathy, a protein also termed megalin [1]. Anti-LRP2 nephropathy is a recently described entity that has features of both tubulointerstitial disease and segmental membranous nephropathy [2, 3]. The originality of the present report consists of the association of a disease thought to be rare (only 13 in prior described patients) with B-cell lymphoproliferative disease. Together with the finding of a third case (already reported in [3]), for a total of 1.3% of 224 native kidney biopsies among persons >65 years, this raises the issue of the underdiagnoses of LRP2 nephropathy, on top of the potential association with B-cell malignancy. We now put these findings in context within the wider frame of autoimmunity against megalin/LRP2 and related antigens such as Fx1A and CD69.

## WHAT IS LRP2?

**FIGURE 1:**
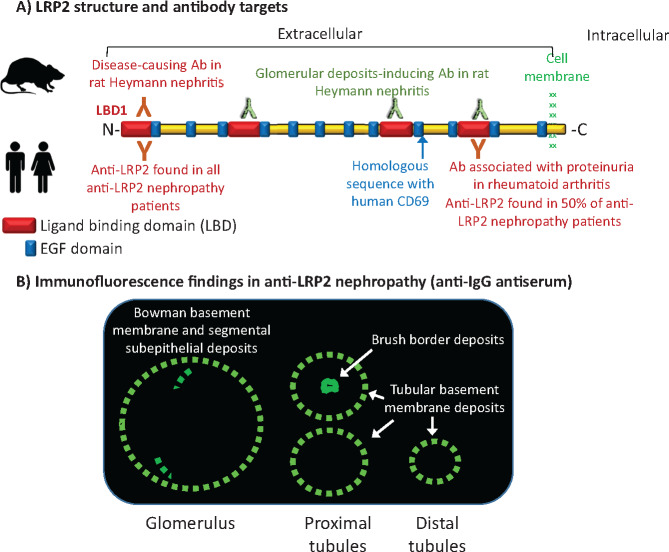
LRP2 and features of LRP2 nephropathy. (**A**) LRP2 is a large (around 4600 amino acids, molecular weight 600 kDa) transmembrane protein containing four LBDs (formed by low-density lipoprotein receptor type A repeats) and multiple EGF domains. In rats, antibodies against any of the LBDs induced glomerular subepithelial deposits, but only those against a specific epitope in LBD1 also induce proteinuria [6]. Human antibodies from all patients with anti-LRP2 nephropathy also bound LBD1, while some sera additionally bound other LBDs, most often LBD4 [2]. LRP2 shares a brief amino acid sequence with CD69 and anti-LRP2 autoantibodies cross-reactive with CD69 were detected in rheumatoid arthritis [7]. Additionally, in rheumatoid arthritis, anti-LRP2 antibodies directed against epitopes in LBD4 were associated with proteinuria [7]. (**B**) Key features of anti-LRP2 nephropathy. By immunofluorescence, anti-LRP2 nephropathy is characterized by segmental glomerular subepithelial IgG deposits, Bowman and tubular basement membrane IgG deposits, and in some cases, proximal tubular cell brush border IgG deposits. EGF: epidermal growth factor, LDL: low density lipoprotein.

LRP2, initially identified as gp330 and also known as megalin, is a brush border of proximal tubular cell transporter that is also expressed by extrarenal cells such as thyroid cells, and, in the kidney, by podocytes [4, 5] (Figure 1A). LRP2 has a key role in the recovery of proteins from the glomerular ultrafiltrate by proximal tubules, and LRP2-deficient mice develop proteinuria. In humans, LRP2 mutations cause the Donnai-Barrow/Facio-oculo-acoustico-renal syndrome, which is characterized, among other things, by low molecular weight proteinuria [8]. LRP2 function in podocytes is less well understood and may be modified by potential binding partners such as MAGI-1 [9]. It was observed that LRP2 contributes to the podocyte uptake of the enzyme replacement therapy compound agalsidase in patients with Fabry disease [5].

## WHAT IS ANTI-LRP2 NEPHROPATHY?

Anti-LRP2 nephropathy is recently proposed name for an autoimmune kidney disease with features of both segmental membranous nephropathy and tubulointerstitial nephropathy characterized by immune deposits in the glomerulus (segmental subepithelial deposits), Bowman capsule and tubules (tubular basement membrane, proximal tubular brush border) associated with circulating anti-LRP2 antibodies [1, 2] (Figure 1B). This is a more specific term and entity than anti-brush border antibody disease, a name that it had received in the past. Additional histological features include segmental podocyte effacement, interstitial inflammation and acute tubular injury. Most patients are >65 years of age and have subnephrotic proteinuria. Although tubular injury was described as acute, around 50% of the patients progressed to end-stage kidney disease (ESRD) and the rest fulfilled the criteria for chronic kidney disease (CKD), having decreased glomerular filtration rate for months [1, 2]. Indeed, the patients reported by Gamayo *et al*. had tubular atrophy and interstitial fibrosis.

Anti-LRP2 nephropathy has features of a mixed glomerular/tubular nephropathy, given that histological glomerular injury most closely resembled segmental membranous nephropathy. This would be expected to cause a mild glomerular-type proteinuria. Proteinuria, in turn, may facilitate access of immunoglobulin G (IgG) to the brush borders of proximal tubular cells. As Gamayo *et al*. suggest, prior cases may have been considered as atypical forms of membranous nephropathy [13].

## HOW IS ANTI-LRP2 NEPHROPATHY DIAGNOSED?

The diagnosis may be suspected by the immunofluorescence finding of IgG and C3 deposition in Bowman capsule and tubular basement membranes, as well as segmental glomerular subepithelial deposits [2] (Figure 1B). In some patients, immunofluorescence is also positive in the brush borders of proximal tubules. Electron microscopy confirms the presence of deposits. Additionally, anti-LRP2 autoimmunity should be demonstrated. Currently, this is best done by immunostaining for LRP2 of the deposits or using immunofluorescence to test patient serum in normal kidney. However, glomerular deposits in LRP2 nephropathy may not stain for LRP2.

## WHY ARE THERE GLOMERULAR SUBEPITHELIAL DEPOSITS IN ANTI-LRP2 NEPHROPATHY?

This is a striking finding that bears upon the history of experimental membranous nephropathy and the old debate on whether podocytes express LRP2.

**FIGURE 2:**
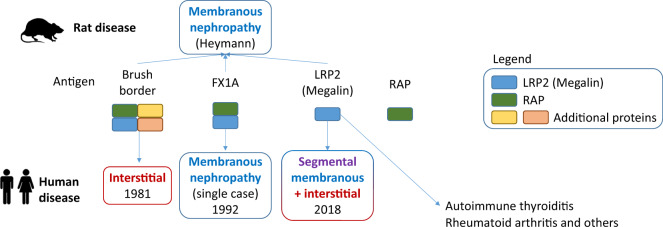
LRP2 and related antigens in the pathogenesis of the human and experimental disease. Immunization with brush border antigens resulted in rat membranous nephropathy (Heymman nephritis). A purified antigen termed Fx1A, composed of LRP2 and receptor-associated protein, also triggered autoimmunity yielding membranous nephropathy in rats. The antigenicity responsible for the kidney disease was traced to LRP2. In humans, serum anti-brush border antibodies were associated with tubulointerstitial disease in the 1980s [11, 12]. A single report associated circulating anti-Fx1A antibodies with human membranous nephropathy [10]. More recently, LRP2 has been identified as the antigen recognized by autoantibodies causing interstitial disease with segmental membranous nephropathy, termed as anti-LRP2 nephropathy [1–3].

Heymann nephritis is a widely used rat model of membranous nephropathy, elicited initially by thrombospondin type-1 domain-containing 7 A (TSHD7A) antigen, called Fx1A. Fx1A is composed of LRP2 and other proteins (Figure 2), but only anti-LRP2 antibodies caused membranous nephropathy in rats [13]. Thus, LRP2 and Fx1A became prime suspects to be the antigenic targets in human idiopathic membranous nephropathy. Indeed, membranous nephropathy characterized by circulating anti-Fx1A antibodies was described in a single patient with hydronephrosis [10]. However, no anti-Fx1A antibodies were found in 12 other membranous nephropathy patients tested. In this regard, it was widely accepted that neither Fx1A nor LRP2 was a key target in human primary membranous nephropathy, because LRP2 was not thought to be expressed by human podocytes. Primary membranous nephropathy is characterized by autoantibodies against podocyte antigens leading to subepithelial immune complex deposition, podocyte injury and proteinuria that may cause nephrotic syndrome, which is treated with immunosuppressive drugs such as rituximab [14, 15]. From 2009, it has been known that most primary membranous nephropathy patients have antibodies to the phospholipase A2 receptor although there are more pathogenic antibodies such as those against TSHD7A [15, 16]. Thus, it is now clearly established that LRP2 or related antigens do not play a role in human primary membranous nephropathy. It is further unlikely that human anti-Fx1A nephropathy represented an early example of anti-LRP2 nephropathy since the biopsy was described as characteristic of membranous nephropathy, and no brush border immune deposits were described despite the presence of subnephrotic proteinuria [10]. However, serum anti-Fx1A antibodies were studied at a different time point than the biopsy. Thus, the kidney histology at the time of the findings of circulating anti-Fx1A antibodies was unknown.

**FIGURE 3:**
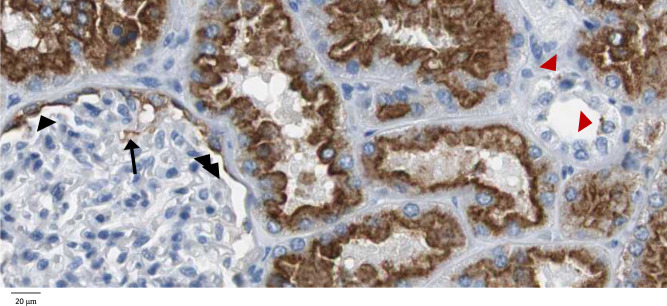
LRP2 expression in normal human kidney according to Human Protein Atlas. Brush border in proximal tubules is intensely stained. Although the Protein Atlas assessed glomerular staining as negative, some parietal epithelial cells were stained in some glomeruli (black arrowheads), and very occasional segmental staining was observed in some glomeruli that could correspond to podocytes (arrow) as well as in distal tubules (red arrowhead). Image credit: Human Protein Atlas, antibody: HPA005980 [19]; https://www.proteinatlas.org/ENSG00000081479-LRP2/tissue/kidney#imgm (accessed 31 October 2019).

There is debate as to whether human podocytes express LRP2 [5, 6, 17, 18]. The lack of human podocyte expression, as opposed to expression by rat podocytes, was considered a reason why anti-LRP2 antibodies induced membranous nephropathy in rats (Heymann nephritis), but not in humans, with the sole potential exception of the single patient with anti-Fx1A antibodies [10, 18]. While any potential LRP2 expression by human podocytes does not match the huge amounts of LRP2 found in the proximal tubular brush border, as once more shown by Gamayo *et al*. in this issue, the finding of podocyte subepithelial deposits in anti-LRP2 nephropathy strongly supports the arguments of the pro-podocyte LRP2 camp [1, 2]. In this regard, human podocyte expression of LRP2 is well documented, both in culture and *in vivo*: there is strong evidence that human-cultured podocytes express LRP2 in the cell surface, as it was eluted as an agalsidase-bound protein and was located to the cell surface by immunofluorescence [5]. Additionally, *LRP2* mRNA was expressed in isolated human glomeruli and immunofluorescence colocalized LRP2 protein with cells expressing the podocyte marker Wilms tumor-1 (WT-1) [5, 6, 17]. The segmental immune deposits in the subepithelial space of the glomerular capillary wall in the present case reports, the Protein Atlas images of segmental anti-LRP2 staining in the glomerular capillary wall (Figure 3) and the demonstration that LRP2 contributes to agalsidase uptake by human podocytes in patients treated for Fabry disease support that podocytes express LRP2 although at lower levels than proximal tubular cells [5]. This may also apply to the tubular basement membrane deposits found in both proximal and distal tubules. They likely represent LRP2 expression in the basolateral membranes of both proximal and distal tubular cells. These thoughts are not anecdotal. Rather, they may lead to a better understanding of the pathogenesis of anti-LRP2 nephropathy. Thus, as pointed out by Gamayo *et al*., LRP2 may have not been found in the capillary wall deposits due to low levels of antigen or to the presence of fragments not recognized by the anti-LRP2 antibody used [1].

Anti-LRP2 nephropathy was not associated with nephrotic syndrome. This may be related to the segmental nature of the glomerular subepithelial deposits or to the observation in rats that only antibodies against certain LRP2 epitopes are associated with proteinuric kidney disease [20] (Figure 1A). However, human anti-LRP2 antibodies reacted with the N-terminal LRP2 domain, as do pathogenic anti-rat LRP2 antibodies [2].

## THE CLINICAL PRESENTATION OF ANTI-LRP2 NEPHROPATHY IS DESCRIBED AS ACUTE KIDNEY INJURY—IS THIS THE CASE?

The original report indicated that all patients presented with acute kidney injury (AKI) [2]. However, from the clinical data provided, the diagnosis of AKI is not obvious (no baseline serum creatinine is provided before the episode) and all patients fulfilled CKD criteria during follow-up. Specifically, two patients had stable renal dysfunction for 6–10 months in the absence of therapy. A further one had spontaneous improvement of AKI, questioning the role of the immune disease in AKI, and renal function remained within the CKD range. Seven patients had underlying disease that may damage the kidney (five were diabetics, one nephrolithiasis and one sarcoidosis). Around 50% progressed to ESRD within 1.5–8 months and one recurred post-transplantation. Some patients progressing to ESRD had been treated with prednisone or rituximab. A patient treated with prednisone/cyclophosphamide had an immunological remission but persisted with CKD.

The course of case reports in this issue of *ckj* is also in line with rapidly progressive CKD rather than with AKI, since creatinine increased over the course of 7–15 months and tubular atrophy and interstitial fibrosis were present [1]. Given this timeline and being proximal tubular cells key targets, Fanconi syndrome or at least tubular proteinuria might have been expected. Specifically, antibodies targeting LRP2 may have interfered with a key transport system to reabsorb filtered proteins, so a tubular pattern of proteinuria may have been expected. This was not described, but it is worth exploring since if present, it may provide diagnostic clues.

## WHAT DRIVES ANTI-LRP2 AUTOIMMUNITY?

**FIGURE 4:**
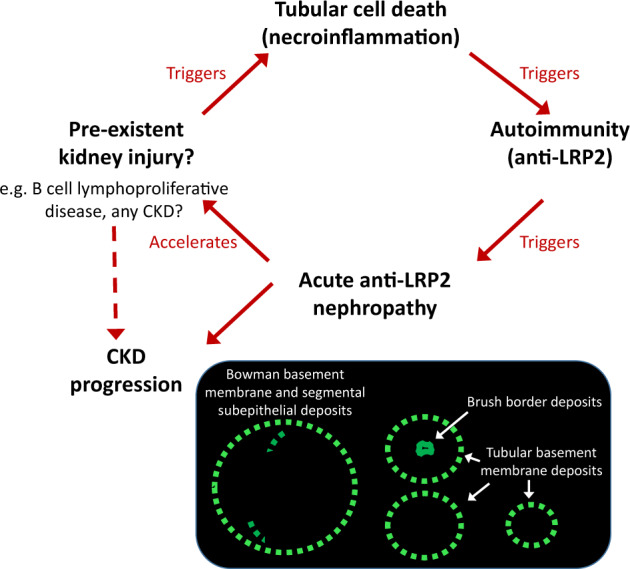
Working hypothesis on the association between B-cell lymphoproliferative and anti-LRP2 nephropathy. Prior kidney injury may trigger regulated necrosis cell death, a source of necroinflammation that may favour immune responses against proximal tubular cell antigens, such as LRP2. This may cause acute anti-LRP2 nephropathy, in which histological evidence of acute kidney injury has been described, but in a context of prior kidney disease, which may explain the concomitant chronic findings in kidney biopsies. Anti-LRP2 nephropathy may in turn accelerate CKD progression or lead itself to CKD.

A common topic may be tubulointerstitial injury. Hydronephrosis, lymphoma infiltration of the kidney, IgG4 plasma cell infiltration, diabetes, nephrolithiasis or sarcoidosis present in case reports [1–3] may cause tubulointerstitial injury with release of autoantigens and priming for an immune response. In this regard, there is accumulating evidence that regulated necrosis is a mode of tubular cell death during kidney injury that is characterized by being immunogenic, triggering the so-called necroinflammation [7, 21]. A working hypothesis might be that kidney injury triggers immunity against LRP2 (Figure 4). Initial deposits may take place in the glomerular and tubular subepithelial space since the large size of IgG may preclude the arrival of large amounts of IgG to the tubular lumen. Given the low amounts of LRP2 in podocytes and in basolateral membranes, this would lead to segmental deposits that do cause podocyte injury, as evidenced by segmental foot process effacement lining the subepithelial deposits. This, in turn, may increase the protein permeability of the glomerular capillary wall, but being injury segmental in nature, proteinuria may remain subnephrotic. Filtering of IgG by some glomeruli may then result in antibody binding to some proximal tubular brush borders. We hypothesize that local triggers may explain the frequent presence of factors associated with kidney injury, the acute presentation despite the evidence of chronicity in the biopsy and the subsequent course as CKD.

## SHOULD ANTI-LRP2 NEPHROPATHY PATIENTS HAVE EXTRARENAL MANIFESTATIONS?

Up to now, no extrarenal features of anti-LRP2 nephropathy have been described and indeed autopsy studies in three patients did not disclose autoimmune disease in other organs, including the thyroid [2]. However, autoantibodies to LRP2 have also been associated with autoimmune thyroiditis and have been observed in 87% of patients with rheumatoid arthritis, 40% of those with systemic lupus erythematous (SLE) and 35% of those with systemic sclerosis, while they were infrequent in other arthritis (15%) and Behçet’s disease (3%) [22, 23]. In this regard, Gamayo *et al*. report hypothyroidism in one patient and in the original report, 4/10 patients had positive or borderline values for other autoantibodies, most commonly antinuclear antibodies [1, 2]. At present, it is unclear what is the significance of anti-LRP2 autoantibodies in rheumatoid arthritis or what determines that different anti-LRP2 antibodies cause damage in different organs. Interestingly, only rheumatoid arthritis patients with certain anti-LRP2 antibodies had a higher prevalence of proteinuria, suggesting a potential role for antibodies with different specificities in determining tissue injury or the distribution of tissue injury. However, the target of these antibodies associated with proteinuria localized to a different LRP2 domain than the antibodies inducing Heymann nephritis in rats or the most common antibodies in anti-LRP2 nephropathy (Figure 1A) [23].

## WHY DO NOT ALL PATIENTS WITH ANTI-LRP2 ANTIBODIES DEVELOP KIDNEY DISEASE?

This is unclear. Autoantibodies to CD69 react to the homologous amino acid sequence in LRP2 (EKNLYWI in CD69 and EKRLYWI in LRP2) [23, 24] (Figure 1A). In rheumatoid arthritis, all anti-CD69-positive samples reacted with LRP2 fragments corresponding to amino acids 3464–4090 near the C-terminal domain in the fourth ligand-binding domain (LBD) of LRP2 [23]. Additionally, anti-LRP2 antibodies and specially those against certain epitopes in the fourth LBD of LRP2 were associated with proteinuria in rheumatoid arthritis but not in SLE [23]. However, no kidney biopsies were reported. This raises the question of whether different forms of anti-LRP2 nephropathy may exist, with some milder forms with preserved renal function being undiagnosed in the context of systemic disease. Alternatively, not all anti-LRP2 antibodies are pathogenic, as shown for rat Heymann nephritis in the past [20]. Thus, in the absence of kidney biopsies, it remains unclear whether circulating autoantibodies in rheumatoid arthritis patients bind to kidney LRP2 or cause kidney disease.

## WHAT ARE THE KEY TAKEAWAYS FROM THE PRESENT REPORT?

Anti-LRP2 nephropathy may be more frequent than previously thought. In 2016, a male was reported with tubulointerstitial nephritis with immune deposits in the tubular basement membrane and glomerular subepithelial deposits [25]. By 2018, 10 patients were reported that shared autoantibodies against the N-terminal portion of LRP2 [2] and there has been a further case so far in 2019 [3]. In this issue of *ckj*, Gamayo *et al*. report two further cases that developed in patients with progressive low-grade B-cell lymphoma characterized by kidney infiltrates [1]. Clinically, there was subnephrotic proteinuria and progressive kidney failure. Histologically, there was lymphoma infiltration, and additionally, tubulointerstitial nephritis, segmental membranous nephropathy and polyclonal IgG and C3 deposits in tubular basement membrane, in proximal tubule brush borders and in subepithelial location in glomeruli. Even after the description of these cases, anti-LRP2 nephropathy remains infrequent and may still be underdiagnosed, so it is key that pathologists become familiar with the characteristics features of this new entity.

## UNANSWERED QUESTIONS

Addressing several issues will further increase our understanding of the disease and its implications. Reports to date have relied on immunofluorescence to detect and monitor circulating anti-LRP2 levels. Development of high-throughput assays that can be integrated into clinical laboratory workstations will provide a better estimation of the frequency of these antibodies and their association with evidence of kidney disease in the general population and in patients with autoimmune or B-cell disease. In this regard, there is some evidence that, as for other autoimmune diseases, immunological remission may precede kidney remission and may guide therapy in anti-LRP2 nephropathy. Since recurrence after transplantation has been described, the impact of such assays on patient management, including timing of transplantation, could be large. However, at present, the specificity and sensitivity of circulating anti-LRP2 antibodies for anti-LRP2 nephropathy remain unknown.

Despite the reporting of AKI as the clinical presentation and of a mainly acute pattern of tubular injury, this does not fit well with the clinical data provided in the reports, which show elevated serum creatinine for months before or after the biopsy. Moreover, some of the biopsy images provided are consistent with chronicity as they show strumoid atrophic tubules and a patchy distribution of nephrons with thickened glomerular and tubular basement membranes. Thus, it is likely that the clinical spectrum is more diverse than reported. In this regard, and given the age range of the patients, it is worth exploring to what extent they had prior CKD, and whether this may be caused by anti-LRP2 nephropathy or whether prior CKD may even have contributed to trigger anti-LRP2 nephropathy through tubular cell death (e.g. necroinflammation) favouring the development of autoimmunity [7] (Figure 4). Is anti-LRP2 disease a cause of low-level proteinuria in the general population and in patients with rheumatoid arthritis? Might it be a cause of low-level proteinuria-associated CKD frequently attributed to hypertensive nephrosclerosis? Is kidney disease part of a wider spectrum of LRP2-associated autoimmunity that may include disease of other organs like the thyroid?

Finally, what is the treatment of anti-LRP2 nephropathy? Outcomes have been so far dismal, with success reported for the cyclophosphamide/corticoids combination in one patient and failure of rituximab in another [2]. Now, Gamayo *et al*. report improvement in serum creatinine and decrease in anti-LRP2 titre following rituximab therapy in one patient [1]. Although a majority of patients were treated with steroids, it is likely that the general principles of therapy will be closer to therapy for primary membranous nephropathy, in which steroids alone are not beneficial. In this regard, the therapeutic paradigm may be closer to membranous nephropathy than to acute tubulointerstitial nephritis, given a closer pathogenesis [15, 25].

## FUNDING

The authors were supported by PI16/02057, PI19/00588, PI19/00815, DTS18/00032, ERA-PerMed-JTC2018 (KIDNEY ATTACK) AC18/00064 and PERSTIGAN AC18/00071, ISCIII-RETIC REDinREN RD016/0009 FEDER funds, Fundacion Renal Iñigo Álvarez de Toledo (FRIAT), Comunidad de Madrid CIFRA2 B2017/BMD-3686. M.V.P.-G. was supported by the Rio Hortega program of ISCIII.

## CONFLICT OF INTEREST STATEMENT

None declared.
